# Performance Optimization and Carbon Reduction Potential of Bamboo Biochar for Lightweight Artificial Aggregates

**DOI:** 10.3390/ma18235415

**Published:** 2025-12-01

**Authors:** Haibao Liu, Lingbao Bu, Yulin Wang, Mingxu Chen, Dongdong Chen

**Affiliations:** 1 School of Hydraulic and Civil Engineering, Ludong University, Yantai 264025, China; 18865620718@163.com (L.B.); m15684230315@163.com (Y.W.); 2 College of Civil Engineering & Architecture, Qingdao Agricultural University, Qingdao 266109, China; chenmx@qau.edu.cn; 3 School of Materials Science and Engineering, Wuhan University of Technology, Wuhan 430070, China

**Keywords:** bamboo biochar, lightweight artificial aggregate, core-shell structure, CO_2_ uptake, carbon footprint

## Abstract

To realize the efficient utilization of biochar and construction solid waste in building materials production, a novel core–shell aggregate concept is proposed, in which artificial aggregates are prepared by encapsulating coarse-particle bamboo biochar (C-BB) with concrete slurry waste (CSW), calcium carbide slag (CCS), and fine-particle bamboo biochar (F-BB). The results showed that the best engineering properties of the artificial aggregates were achieved when the F-BB content was about 6%, with crushing strength, water absorption, and bulk density values of 4.7 MPa, 14.3%, and 796 kg/m^3^, respectively. In addition, the artificial aggregates have promising potential for CO_2_ uptake under a CO_2_ curing system and can achieve 5.72% (by mass) uptake when the F-BB content is 6%. This performance is attributed to the formation of well-developed CO_2_ transport channels in the shell matrix by the F-BB particles. In summary, the novel core–shell aggregate not only has better engineering properties than commercial lightweight aggregates but also offers significant potential for CO_2_ sequestration, opening new opportunities for the efficient application of biochar in construction materials with both engineering and environmental benefits.

## 1. Introduction

High CO_2_ emissions from the production of construction materials are one of the main problems hindering the sustainable development of the construction industry [1,2]. According to government reports, the total carbon emissions of China’s construction industry exceeded 5 billion tons of CO_2_ in 2023, accounting for 48% of the country’s total carbon emissions [3,4]. On the one hand, the massive exploitation of natural aggregates and raw materials for cement production has negatively affected the ecological environment and biodiversity. On the other hand, the open storage of construction waste poses a serious threat to environmental safety. Therefore, the development of recycled aggregate production technology that combines construction waste utilization with carbon emission reduction is of great significance to the sustainable development of the construction industry and to the realization of the Chinese government’s current goal of “carbon peaking and carbon neutrality” [5,6].

In addition to direct air capture and carbon capture and storage, biochar sequestration is also recognized by the Intergovernmental Panel on Climate Change (IPCC) as a method of carbon sequestration, which gives biochar great potential for reducing carbon emissions by replacing high-carbon-emitting building materials with cleaner production in the construction industry [7,8]. Biochar is prepared by pyrolyzing organic matter such as agricultural and forestry wastes, manure, and household waste under slow-heating conditions. It is a carbon-rich product that effectively captures atmospheric carbon dioxide absorbed through plant photosynthesis. According to studies, each ton of biochar produced can achieve more than 2 tons of CO_2_ sequestration equivalent. Compared with mineral aggregates, the low density and high porosity of biochar have the potential to produce lightweight concrete. In recent years, many studies have been carried out on the use of biochar as a substitute for binder materials in the production of lightweight artificial aggregates and lightweight concrete [9,10]. Lin and Aneja et al. [11,12] utilized different types of biochar to produce concrete by substituting binder materials in equal mass proportions, thereby realizing the potential of agricultural solid waste recycling and the green production of concrete. However, the high dosage of biochar is inevitably detrimental to the engineering properties of concrete. Jia [13] used municipal solid waste biochar to replace cement in equal mass proportions to prepare concrete, and the results show that when the dosage of biochar is higher than 10%, the mechanical properties of the concrete are impaired to varying degrees. However, it has positive significance for improving the frost resistance of concrete. Alexey [14] analyzed that the addition of a 6% mass ratio of rice straw biochar can improve the compressive strength of concrete and increase the modulus of elasticity by 20% and 14%, respectively, and this improvement is attributed to the internal curing effect of adsorbed water within the biochar.

Song et al. [15] attempted to prepare foam concrete using rice hull biochar obtained by pyrolysis at 700 °C, and the results showed that a 5% admixture of biochar can improve the mechanical properties of concrete. The results also showed that rice husk biochar at a 5% admixture could refine the pore structure, improve the surface morphology of the foam concrete, and increase the stability of the foam inside the concrete. Chen et al. [16] used a mass fraction of 30% biochar instead of minerals for the production of concrete, achieving sequestration of more than 130 kg CO_2_ equivalents per ton of concrete. However, it also had a somewhat negative impact on mechanical properties. In addition, Zhang [17] attempted to prepare ultra-high-performance concrete (UHPC) using walnut shell biochar to replace river sand. The porosity of UHPC increased by 1.6 times when the biochar substitution rate was 1% compared with the control group, while the compressive strength decreased by more than 5%. The above experiments show that when biochar is used directly to replace binder materials in the preparation of concrete, and the substitution rate exceeds a reasonable range, the mechanical properties of the concrete inevitably deteriorate, leading to a significant reduction in the potential for the application of biochar in the field of concrete. In addition, the degree of solidity of biochar particles varies greatly depending on the density of the structural matrix of the raw material, which also leads to uneven engineering properties of concrete.

The use of artificial aggregates is an ideal substitute for natural aggregates in concrete production. Compared with the dense aggregate matrix of natural aggregates, artificial aggregates have good processability. Typically, porous materials are introduced during the preparation of synthetic aggregates to produce lightweight synthetic aggregates. This characteristic not only gives lightweight synthetic aggregate concrete excellent thermal properties but also significantly reduces the carbon footprint of concrete structures over their service life [18,19]. However, commercial lightweight aggregates are often prepared using processes such as calcination, which significantly reduce their environmental cleanliness, making conventional lightweight aggregates less competitive in today’s global warming climate due to greenhouse gas emissions. In contrast, cold-bonded aggregates prepared at ambient temperatures are receiving increasing attention from building material manufacturers because of their advantages of low cost, low energy consumption, and high environmental friendliness [20,21,22]. Chen [23] explored the effect of biochar pyrolysis temperature on the engineering properties of artificial aggregates prepared with biochar. It was found that increasing the pyrolysis temperature of biochar can effectively improve the properties of artificial aggregates, and the compressive strength and density of artificial aggregate concrete at 28 curing days reached 57.8 MPa and 1.77 g·cm^−3^, respectively, which meet the requirements of high-strength lightweight aggregate concrete. Jiang [24] actively attempted to prepare permeable bricks using woody biochar as a mineral filler substitute. It was found that the elongation and evaporation capacity of permeable bricks prepared with a 50% biochar substitution rate met the requirements, and the capillary water absorption increased from 0.364 to 1.108 kg/m^2^h^0.5^. Liu et al. [25] used sawdust biochar combined with bottom ash from municipal waste incineration to prepare lightweight synthetic aggregates and investigated the carbon sequestration capacity of the synthetic aggregates under the CO_2_ curing system. The results showed that a maximum of 26.67 kg/ton of CO_2_ sequestration could be achieved at a biochar incorporation rate of 5%. Zou et al. [26,27] developed a lightweight artificial aggregate with a loose bulk density of 789 kg/m^3^, a compressive strength of 6.84 MPa, and a water absorption rate of 19.4% by using corn cob biochar. In addition, the carbon dioxide emission from producing one ton of artificial aggregate is −69 kg, which is much lower than that of commercial sintered artificial aggregates.

The above studies show that lightweight artificial aggregates with the required properties can be prepared using a reasonable proportion of biochar. However, when the dosage exceeds a certain threshold, the low matrix strength and high water absorption characteristics of biochar particles result in a steep decline in aggregate matrix compactness, which significantly limits the amount of biochar that can be used in artificial aggregates. In addition, according to the accounting method of the IPCC, artificial aggregates prepared with biochar can sequester an equivalent amount of carbon dioxide. The huge amount of carbon dioxide unavoidably emitted by human production processes (e.g., power generation from coal-fired plants and the operation of internal combustion engines) has a direct and significant impact on climate change due to current technological limitations. The abundance of calcium hydroxide in the binder matrix of artificial aggregates gives them great potential for carbon dioxide sequestration, and how to utilize this carbon sequestration potential is one of the key technologies that needs to be improved in the preparation of artificial aggregates.

Based on the urgent problems that need to be solved in commercial artificial aggregates, this study innovatively developed core–shell artificial aggregates using the porous characteristics of bamboo biochar (BB). In this concept, coarse-particle biochar (C-BB) is sealed inside the artificial aggregate as a porous lightweight material. This approach not only forms a structurally stable shell structure but also improves the utilization rate of biochar in building materials. Meanwhile, to fully release the CO_2_ uptake potential of cement-based shell materials, a corresponding proportion of fine-particle bamboo biochar (F-BB) was added to the binder materials to form a connected carbon dioxide transmission channel in the shell matrix, thereby achieving effective uptake and storage of carbon dioxide. In addition, to further reduce the carbon footprint of artificial aggregates, calcium carbide slag (CCS) and concrete slurry waste (CSW) were used in place of cementitious materials for the preparation of artificial aggregates.

In this study, the engineering properties of artificial aggregates, including crushing strength, water absorption, and loose bulk density, were investigated. The hydration properties and carbonation degree of different groups of artificial aggregates were characterized by heat of hydration, X-ray diffraction (XRD), and carbon dioxide absorption. In addition, the internal micro-morphology and pore distribution of artificial aggregates were analyzed using scanning electron microscopy (SEM) and X-ray computed tomography (X-CT). Finally, the carbon footprints of core–shell artificial aggregates and common commercial sintered ceramic aggregates were comprehensively evaluated and compared.

## 2. Materials and Preparation Methods

### 2.1. Raw Materials

**Figure 1:**
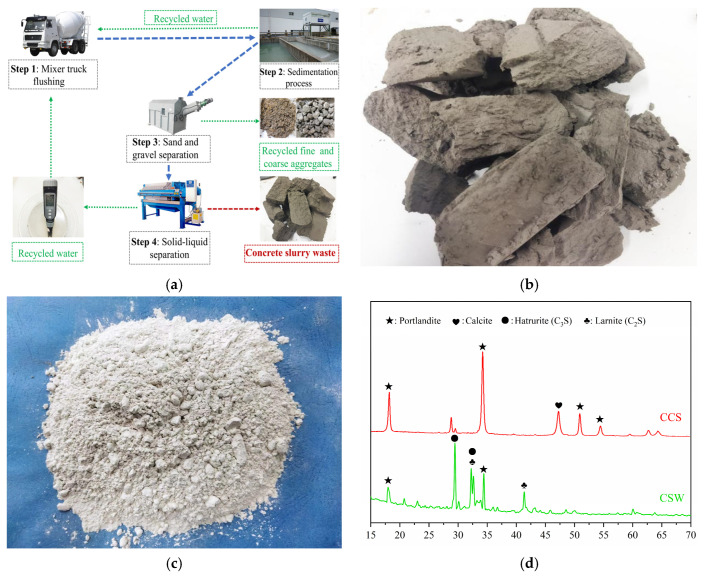
(**a**) CSW preparation process, (**b**) CSW sample, (**c**) CCS sample, and (**d**) binder material chemical composition.

The raw materials used in this study include CSW, CCS, and BB. Ready-mixed concrete plants produce a considerable amount of fresh waste concrete every year due to construction delays, overbooking, and production losses. The CSW used in the experiments was produced from a ready-mixed concrete production line in Yantai, Shandong Province. After the collection, resting, and filter-pressing processes shown in Figure 1a, very low water content lump CSW was obtained (Figure 1b), which was then ground into powder form to be used as a binder material for aggregate pelletizing [28]. The binder materials for the original concrete consisted of ground granulated blast furnace slag and P·O 42.5 cement in the proportion of 20% and 80%. The total time from the ready-mixed concrete mixing process to the collection of CSW was calculated to be 3 h. In addition, to further reduce the carbon footprint of the lightweight artificial aggregate preparation process, the CSW was partially replaced by CCS (Figure 1c), and the chemical compositions of the above binder materials are shown in Figure 1d and Table 1.

The BB used in the experiment was produced by pyrolysis of bamboo from a furniture processing plant, which was heated at a rate of 10 °C/min to 600 °C for 120 min with a residence time of 60 min. Before pyrolysis, the waste bamboo was split, screened, and dried, then crushed into granules with particle sizes ranging from 3 to 6 mm. Compared with other types of biochar prepared by pyrolysis of agricultural straw and wood, BB is structurally stable and has a large number of connected pores of uniform size, which is attributed to the dense matrix organization of bamboo and the abundance of bamboo fibers, endowing BB particles with excellent processability. In addition, the Mohs hardness of the BB matrix was determined to be 3.3, which means that the shell structure matrix doped with BB particles possesses excellent structural stability.

**Figure 2:**
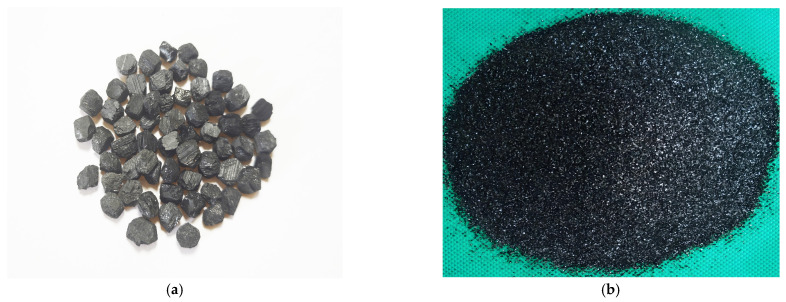

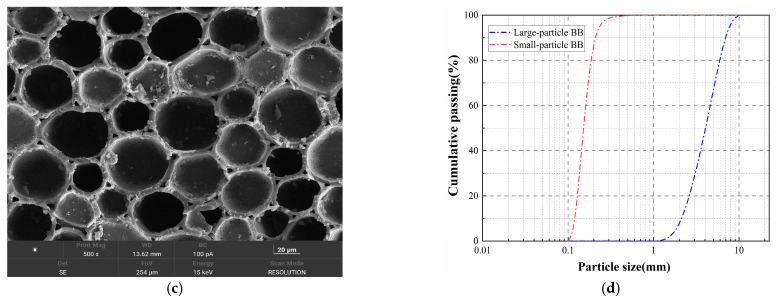
(**a**) C-BB, (**b**) F-BB, (**c**) BB pore microstructure, and (**d**) BB particle size distribution curve.

To reduce the risk of stress-concentration damage of the core–shell artificial aggregates prepared with flake BB particles as core material, the coarse-particle biochar was milled in spherical ink equipment using ultra-lightweight grinding beads for 5 min, producing BB particles with smooth surfaces and few edges (Figure 2a). In addition, some of the large-particle bamboo biochar was crushed and sieved to obtain F-BB with particle sizes ranging from 150 to 250 μm (Figure 2b). As shown in Figure 2c, the BB matrix had a dense structure and was internally covered with a large number of interconnected pores with a pore size range of 20–70 μm. The bulk density and water absorption of the biochar were determined to be 252 kg/m^3^ and 241% (by mass), respectively.

### 2.2. Mix Proportions

The mix proportions of the artificial aggregates are shown in Table 2, where 3–6 mm C-BB was used as the aggregate core material and F-BB was used to replace the binder material at proportions of 2%, 4%, 6%, and 8% by mass in the experiment. In addition, to enhance the CO_2_ uptake potential of the artificial aggregates under a CO_2_ curing system, calcium-hydroxide-rich CCS was used to prepare the artificial aggregates by replacing CSW at a proportion of 10% (by mass). Due to the high porosity of BB particles, which results in high water absorption, additional water was added during the aggregate preparation process to maintain the effective water–cement ratio of the artificial aggregates. The water-to-binder ratio (W/B) used for the shell material was determined to be 0.22 based on continuous attempts during the preparation process.

### 2.3. Preparation Method

In the pelletization process, the binder material (CSW) and BB pellets (with or without F-BB and CCS) were first stirred in a mixer at a rate of 25 rpm for 5 min until well mixed. The technical specifications of the disk granulator were set at a tilt angle of 42 degrees and a rotation speed of 40 rpm. After several attempts of experiments, it was found that the core material (C-BB) was first added in the pelletizing experiment and then uniformly sprayed with water until its surface was fully wetted and then the binder material was added, and after the cementing material was completely wrapped on the surface of the core material, then the above operation could be repeated to obtain the structurally intact core–shell artificial aggregates. It is found that the above production method can effectively reduce the adhesion between the particles of the core material, which in turn improves the preparation efficiency of the core–shell artificial aggregate. After the pelletizing process was completed the rotary pelletizer continued to run for 3 min so that the compaction of the fresh lightweight synthetic aggregate shell matrix was completed. After rotary granulation, the fresh lightweight synthetic aggregates were transferred to different curing environments for curing.

**Figure 3:**
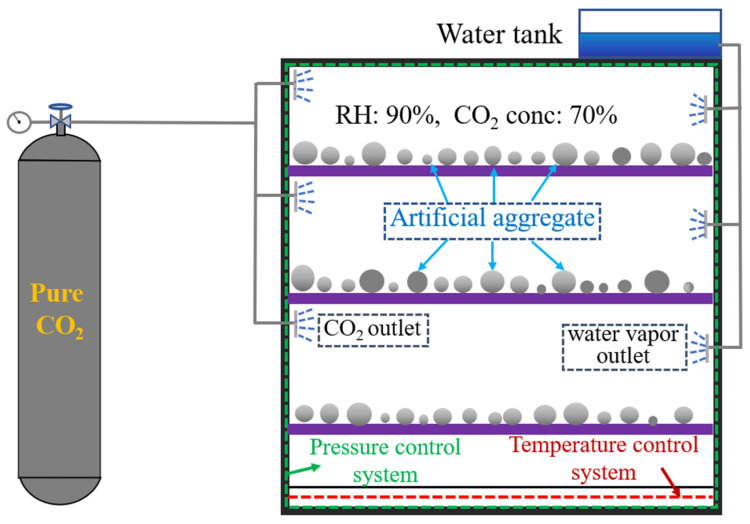
Schematic diagram of artificial aggregates in CC system.

The artificial aggregate curing system includes the AC system and the CC system. In the AC system, fresh aggregates are cured in an environment with 90% relative humidity and 20 °C for 30 days. In the CC system, to improve the carbonation efficiency, the fresh aggregates were cured in an environment with 90% relative humidity, 20 °C, 70% CO_2_ concentration, and 0.15 MPa pressure for 30 days (Figure 3).

### 2.4. Testing Methods

#### 2.4.1. Engineering Properties of Artificial Aggregates

After the granulation process, the fresh artificial aggregates were transferred to the corresponding curing system for maintenance. After curing, 30 randomly selected artificial aggregates in each group were tested for strength, and the average value was taken as the crushing strength in that group. The crushing strength σ was calculated by the following formula [19,29]:

(1) $$\sigma =\frac{2.8P}{\pi d^{2}}$$

where *P* is the peak force under compressive failure (N), and *d* is the distance between loading points (mm). The water absorption rate and bulk density of the artificial aggregates were determined according to the relevant methods specified in Chinese Standard GB/T 17431.2 [19]. To determine the intrusion depth of carbon dioxide gas in artificial aggregates under the CC system, phenolphthalein solution with a solute mass fraction of 5% was sprayed on the cross-section to observe the carbonation depth. In addition, the X-CT method was used to determine the pore distribution of the artificial aggregate shell matrix.

#### 2.4.2. Hydration Heat

A TAM Air microcalorimeter (DE, Newcastle, WY, USA) was used to determine the exothermic pattern of the hydration reaction of binder materials containing CCS and different contents of F-BB by the adiabatic method. Five grams of powdered material were taken and thoroughly mixed with water for 2 min, and then immediately transferred to a well-sealed calorimeter.

#### 2.4.3. XRD

The mineralogical composition of the cement hydration reaction was examined using an X-ray diffractometer (D9 Advanced). The operating conditions of the instrument were as follows: copper target, voltage 45 kV, current 50 mA, scanning range 10–70°, and scanning step 0.02. The shell material samples were first crushed and immersed in isopropyl acetone for 72 h to prevent subsequent hydration reactions. Subsequently, the samples were dried and fully ground, and the powder samples for testing were screened using a 200-mesh aperture sieve.

#### 2.4.4. SEM/X-CT

The internal microstructural observations of aggregates were carried out by scanning electron microscopy (SEM) to analyze the effects of CCS and F-BB at different contents on the microstructure of the aggregates. The samples were soaked in isopropyl alcohol for 72 h to stop the hydration reaction and then vacuum dried at 25 °C for 10 h. The samples were subsequently crushed so that the aggregates could be used for microstructural analysis. Afterwards, the artificial aggregates were crushed to expose the section to be measured and immediately embedded in epoxy resin to prevent oxidation at the section site. To improve the accuracy of the observations, the surfaces were sanded with sandpaper of 600 and 1200 grit to obtain a smooth finish. A TESCAN MIRA LMS scanning electron microscope was used to observe the internal microstructure of the artificial aggregates.

In addition, to observe the pore size and distribution characteristics of the internal pores of the artificial aggregates, a Zeiss METROTOM 800 X-ray CT machine was used. The accelerating voltage was 225 kV, the tube current was 3000 µA, and the scanning resolution was configured to a voxel size of 4 µm.

#### 2.4.5. Calculation of CO_2_ Uptake

The structural part of the aggregate shell was crushed and ground to powder form, and then tested for loss on ignition in a nitrogen atmosphere. In the test, each group of aggregates consisted of two samples: one sample was heated to a constant weight after gradual warming to 550 °C, and the other sample was heated to a constant weight after gradual warming to 900 °C. The CO_2_ uptake of the artificial aggregate shell material was calculated as the difference in the loss on ignition between the two samples [19,30]. It is worth noting that CCS contains a 25% mass fraction of calcium carbonate, which must be excluded from the calculation of CO_2_ uptake to avoid interference in the results. The CO_2_ uptake rate was calculated using Equation (2) [19].

(2) $$\eta_{{CO}_{2}}=(\frac{\Delta M_{\left(550–900\right){}^{\circ}C}}{\Delta M_{d}}-\eta_{SRM})\times 100\%$$

where $\Delta M_{(550–900){}^{\circ}C}$ is the total loss on ignition of calcium carbonate in the sample shell structure, $\Delta M_{d}$ represents the mass of the artificial aggregate shell material sample, $\eta_{CO_{2}}$ denotes the CO_2_ uptake per unit mass of the sample under the CC system, and $\eta_{\mathrm{SRM}}$ represents the amount of CO_2_ contained in the raw shell material.

## 3. Results and Discussion

### 3.1. Hydration Heat Characteristics of Shell Materials

**Figure 4:**
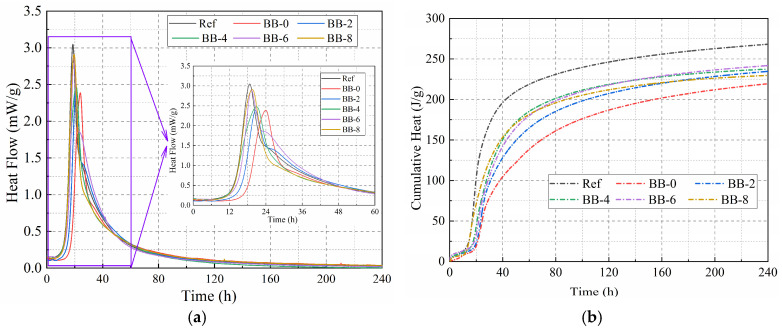
(**a**) The hydration exothermic curves and (**b**) cumulative exothermic curves of the binder materials.

In this study, lightweight artificial aggregates were prepared using CSW, CCS, and specific proportions of F-BB as shell materials at low W/B. Therefore, the influence of the type and amount of shell material on the hydration characteristics was investigated. Figure 4a,b show the hydration exothermic curves and cumulative exothermic curves of the shell materials with a hydration reaction time of 240 h, respectively. The results show that CCS not only slowed down the hydration reaction rate of CSW but also caused a significant decrease in the cumulative heat of hydration. The cumulative heat of hydration for the BB-0 group at 3 and 10 d was 153 J/g and 219 J/g, respectively, which was lower than that of the Ref group. According to Figure 1d, CCS and the hydration products of Portland cement contain a large amount of calcium hydroxide, and the incorporation of CCS hinders the hydration reaction of residual tricalcium silicate (C_3_S) and dicalcium silicate (C_2_S) in CSW, which results in the decrease in the exothermic rate and cumulative heat value. In addition, F-BB effectively eliminated the negative effects of CCS on the hydration reaction rate and cumulative heat of hydration of the binder materials. The 10 d cumulative heat of hydration values of the binder materials were 234.7 J/g, 237.4 J/g, 241 J/g and 229.7 J/g when the F-BB content was 2%, 4%, 6%, and 8%, respectively, which were all higher than that of the BB-0 group at 219 J/g. According to a related report [31], the protrusions on the surface of BB microparticles act as “nucleation” sites during the hydration of mortar, accelerating the growth of hydration products such as hydrated calcium silicate and improving the hydration reaction rate. Furthermore, the numerous pores on the surface of BB particles provide additional space for the growth of hydration products, which improves the degree of hydration reaction and, to some extent, counteracts the negative effect of CCS on the hydration reaction [32,33].

### 3.2. Analysis of Hydration Products

**Figure 5:**
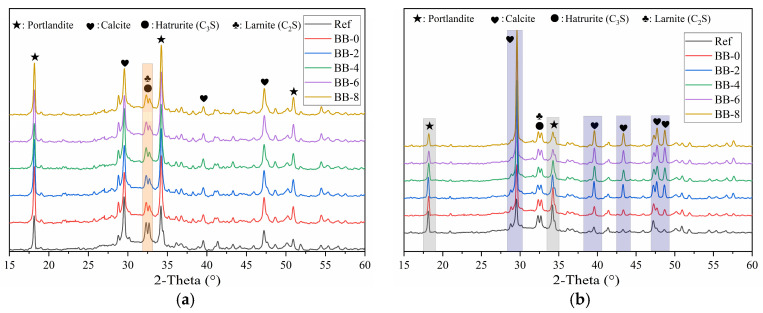
XRD patterns of shell material hydration products under (**a**) AC system and (**b**) CC system.

Figure 5a shows the XRD test results of the shell materials of artificial aggregates cured for 30 d under the AC system. The results show that a certain percentage of unhydrated C_3_S and C_2_S remained, which implies that under the low W/B strategy of artificial aggregates (W/B = 0.22), the participation of unhydrated cement particles in hydration was insufficient. In contrast, F-BB promoted the hydration process of the binder materials, and this effect became more significant with increasing F-BB content. As shown in Table 1, every 2% increase in F-BB content required an additional 25 kg of mixing water, which was adsorbed in the internal pores of F-BB and effectively improved the hydration of CSW through a slow-release effect during the curing period of the artificial aggregates. In addition, as shown in Figure 5b, the C_3_S, C_2_S and calcium hydroxide content decreased under the CC system, which indicated that the carbonation reaction consumed a large amount of calcium hydroxide, thereby further improving the degree of CSW hydration.

### 3.3. Engineering Properties of Artificial Aggregates

#### 3.3.1. Loose Bulk Density

**Figure 6:**
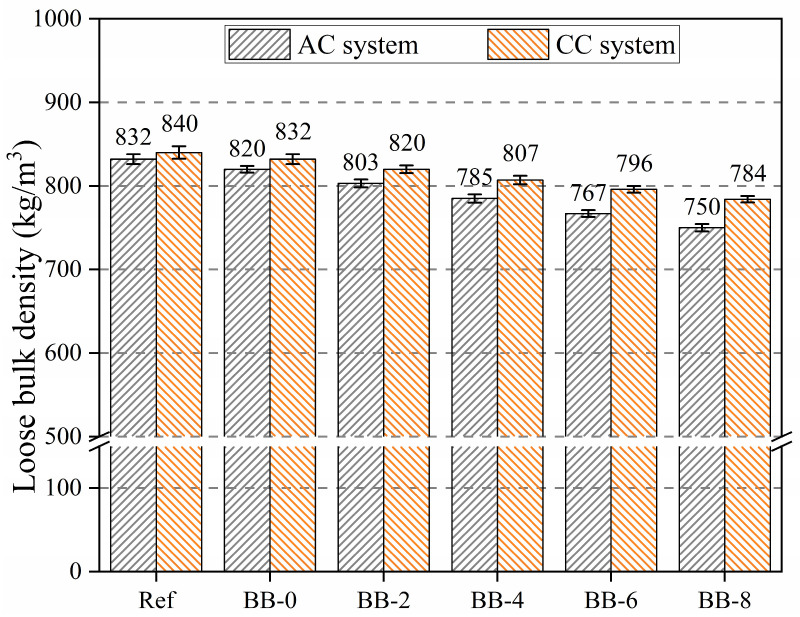
Loose bulk density of artificial aggregates under different curing systems.

As shown in Figure 6, under the CC system, the loose bulk density of artificial aggregates gradually decreased with increasing F-BB content. Compared with 820 kg/m^3^ in the control group, the loose bulk density of aggregates decreased to 803 kg/m^3^, 785 kg/m^3^, 767 kg/m^3^, and 750 kg/m^3^ when the F-BB content was 2%, 4%, 6%, and 8%, respectively. All values met the technical requirements of lightweight artificial aggregates [19]. As shown in Figure 2c, the connected pores inside the BB particles formed a large number of closed pores in the shell matrix after the fresh aggregates hardened, leading to a decrease in loose bulk density. In addition, at 2%, 4%, 6% and 8% BB content, the loose bulk density of aggregates in the CC curing system was 820 kg/m^3^, 807 kg/m^3^, 796 kg/m^3^, and 784 kg/m^3^, respectively. These values increased by 17 kg/m^3^, 22 kg/m^3^, 29 kg/m^3^, and 34 kg/m^3^, respectively, compared with those in the AC system. Under the CC system, a large amount of calcium hydroxide crystals in the hydration products and CCS underwent carbonation, and the resulting calcium carbonate effectively enhanced CO_2_ uptake, leading to an increase in the bulk density of aggregates. In addition, the higher the F-BB content, the greater the increase in loose bulk density of aggregate under the CC system. This phenomenon was attributed to the formation of gas transport channels between the numerous BB particles, which led to a more complete carbonation reaction of calcium hydroxide crystals within the aggregates. The XRD and SEM test results confirmed this conclusion.

#### 3.3.2. Crushing Strength

**Figure 7:**
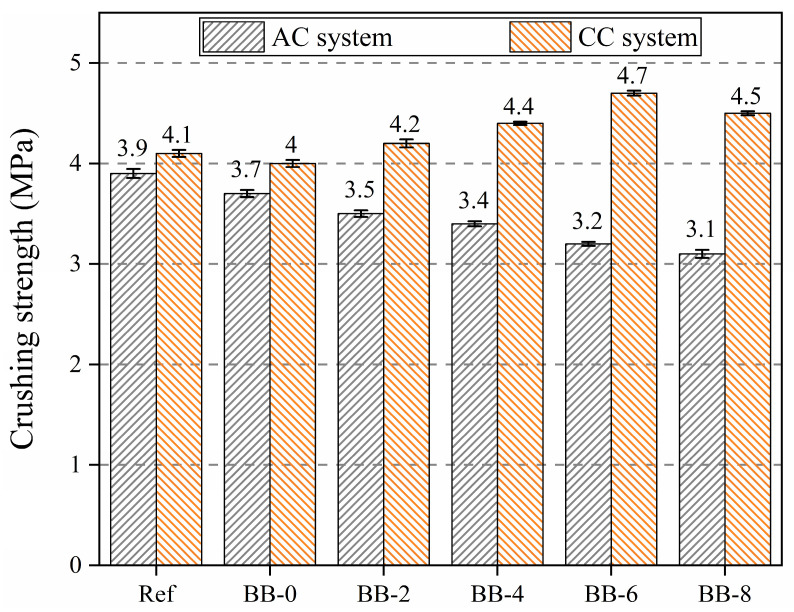
Crushing strength of artificial aggregates under different curing systems.

Figure 7 shows that the addition of CCS and F-BB weakened the crushing strength of the aggregates to different degrees, and the compressive strength of the aggregates reached 3.1 MPa when the BB content was 8%, which indicates that the addition of CCS and F-BB negatively affected the crushing strength of the artificial aggregates.

First, CCS lacks cementing ability, and its particle size is larger than that of cement particles. Therefore, replacing CSW with CCS weakened the densification of the shell material matrix. Second, cement particles such as C_3_S and C_2_S in CSW still have high hydration activity, and they react with water to produce hydrated calcium silicate gel and calcium hydroxide crystals. In contrast, the large amount of calcium hydroxide crystals in CCS affected the degree of hydration of cement particles, which in turn weakened the crushing strength of the aggregates. Third, similar to CCS, BB also lacks cementing ability, and F-BB formed a large number of ITZs in the shell material matrix, further reducing the matrix compactness and leading to a reduction in aggregate crushing strength [34].

**Figure 8:**
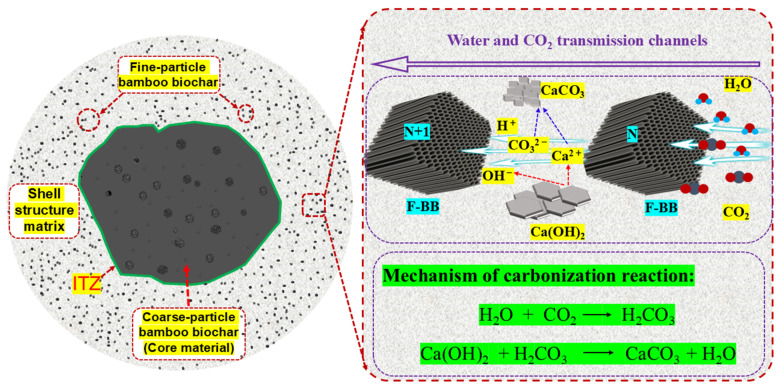
Schematic diagram of CO_2_ transport channels within the shell matrix doped with F-BB.

Unlike the trend of aggregate crushing strength under the AC system, the aggregate compressive strength showed a trend of increasing and then decreasing with the increase in BB particle content under the CC system. The crushing strength of the aggregates reached 4.7 MPa when the F-BB content was 6%. The calcium hydroxide crystals contained in CCS and cement hydration products have great potential for carbon mineralization, and the addition of BB particles made the carbon mineralization reaction easier to achieve. As shown in Figure 8, a large number of F-BB particles formed nearly connected carbon dioxide transport channels in the shell matrix, enabling the calcium hydroxide crystals in the shell matrix to undergo carbonation with carbon dioxide. This process consumed a large number of large-sized calcium hydroxide crystals and generated compact calcium carbonate clusters, thereby effectively improving the compactness of the shell matrix [35]. However, when the BB content exceeded 6%, the aggregate crushing strength decreased, indicating that the excessively high content of F-BB formed a large number of ITZs in the aggregate shell matrix, and its negative effect counteracted the improvement provided by the carbon mineralization reaction.

#### 3.3.3. Water Absorption

**Figure 9:**
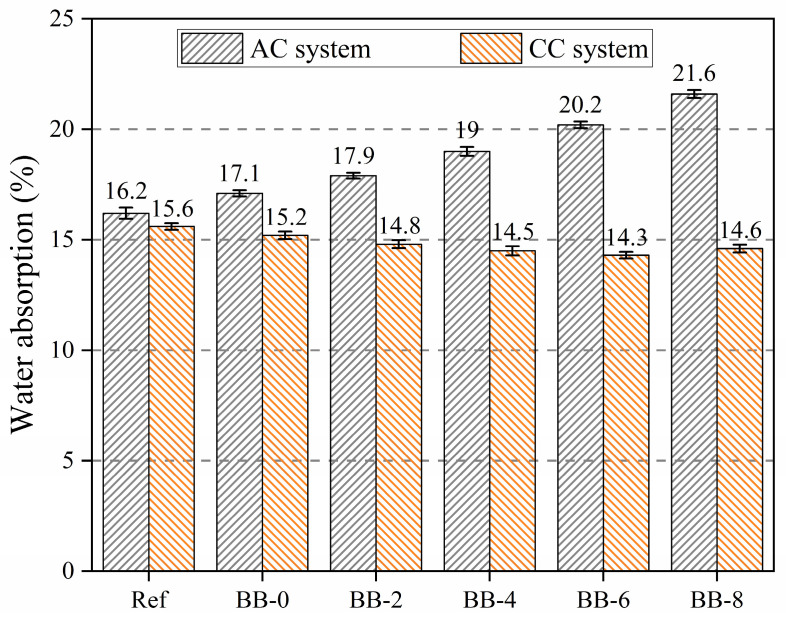
Water absorption of artificial aggregates under different curing system.

Water absorption, similar to crushing strength, is one of the important engineering properties that reflect the degree of compactness of the artificial aggregate shell matrix. As shown in Figure 9, the water absorption of artificial aggregates increased with increasing F-BB content in the AC system, while it decreased with increasing F-BB content in the CC system. This trend was consistent with the change in the crushing strength of the aggregates.

The addition of F-BB weakened the densification of the aggregate shell matrix; however, the connecting pores inside F-BB created favorable conditions for the carbonation reaction in the shell matrix. The calcium carbonate generated by this reaction effectively filled the cracks and pores in the shell matrix, thereby mitigating the deterioration of densification caused by F-BB.

#### 3.3.4. CO_2_ Uptake

**Figure 10:**
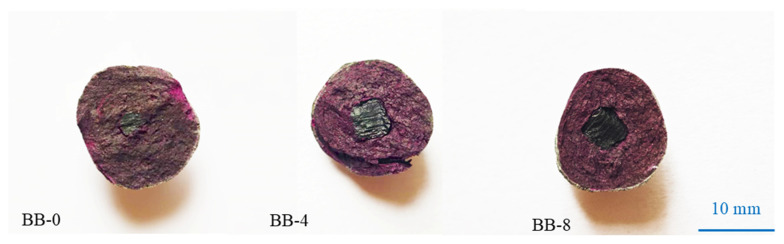
Cross-section coloration of artificial aggregates under AC system.

Figure 10 shows the degree of coloration of the aggregate cross-section under the AC system, and the results indicate that the cross-sections were significantly colored in all groups. Compared with the Ref group, the incorporation of CCS and F-BB had little effect on the carbon dioxide uptake of the artificial aggregates, which means that the trace amount of carbon dioxide in the air did not penetrate effectively into the interior of the aggregates, and only the surface portion underwent carbonation.

**Figure 11:**
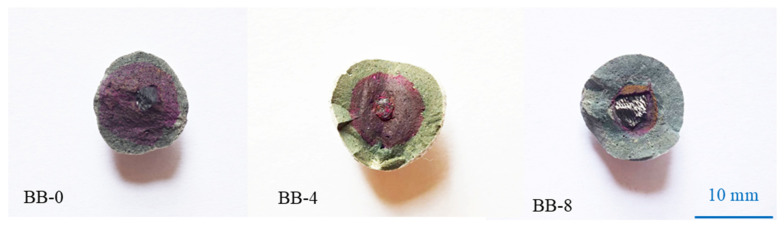
Cross-section coloration of artificial aggregates under CC system.

As shown in Figure 11, compared with the AC system, the surface portion of the Ref group aggregates under the CC system showed significant carbonation, while the core portion still exhibited noticeable coloration. The high concentration of carbon dioxide and the ambient pressure allowed the molecules to effectively penetrate into the interior of the aggregates, while the calcium carbonate clusters generated by the reaction filled the shell matrix and blocked the transport channels, thereby reducing the possibility of further carbonation inside the aggregates. It is worth noting that the incorporation of F-BB accelerated the carbonation reaction inside the aggregates. When the F-BB content was 8%, the coloring of the shell matrix was not obvious, which was attributed to the formation of carbon dioxide transmission channels in the shell matrix by the connected pores of F-BB particles. These channels allowed the carbon dioxide to penetrate effectively into the aggregates, resulting in thorough carbonation even at the core of the shell.

**Figure 12:**
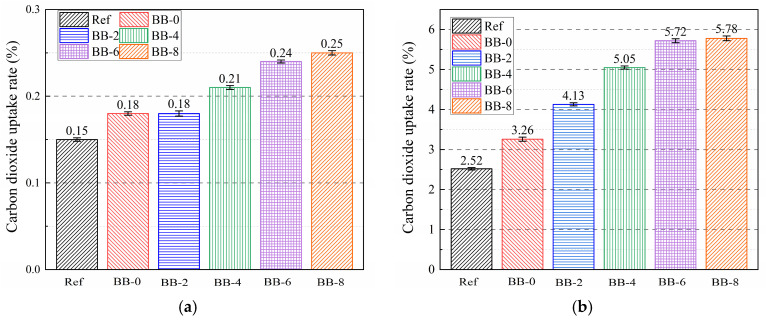
Carbon dioxide uptake rate of aggregate under (**a**) AC curing system and (**b**) CC curing system.

Compared with the AC system, the engineering properties and CO_2_ uptake capacity of artificial aggregates under the CC system are effectively improved, which is of positive significance in the current context of global warming. It is well known that the main carbonation product of the silicate cement hydration system is calcite, which, according to previous studies [19,30,35], decomposes and generates calcium oxide and carbon dioxide in the temperature interval of 550–900 °C. The mass loss from pyrolysis corresponds to the carbon dioxide uptake per unit mass of shell material. Figure 12a shows that under the AC system, the incorporation of CCS had little effect on improving carbon dioxide uptake efficiency, and the uptake rate of artificial aggregates in all groups was lower than 0.5%. This result is consistent with the coloration of the artificial aggregate cross-section shown in Figure 10.

Under the CC system, compared with 2.52% in the Ref group, the carbon dioxide uptake rate of BB-0 increased to 3.26%, which was attributed to the fact that CCS provided sufficient calcium hydroxide for the carbonation reaction. However, the carbonation reaction caused the aggregate surface matrix to become denser and blocked carbon dioxide transport to the core, preventing full utilization of the uptake potential. Consistent with the coloring of the aggregate cross sections, the carbon dioxide uptake rate of the aggregates gradually increased with increasing F-BB content. The uptake rate per unit mass of aggregate increased to 4.13%, 5.05%, 5.72%, and 5.78% when the F-BB content was 2%, 4%, 6%, and 8%, respectively.

### 3.4. SEM/X-CT

**Figure 13:**
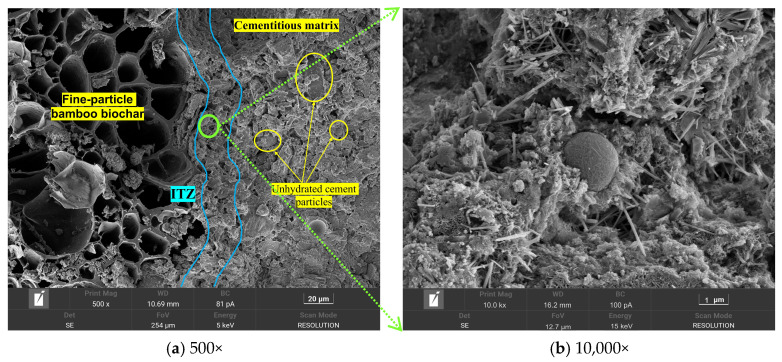
Microstructure morphology of the ITZ between the BB and the shell matrix under AC system.

Figure 13 shows the microstructural morphology of the BB-4 group of artificial aggregates under different curing systems. The results show that the aggregate shell structure, especially the interfacial transition zone (ITZ) between BB particles and the shell matrix, was loose and contained large pores under the AC system. This was attributed to insufficient compaction of the biochar and shell structure with the binder materials during the granulation process.

**Figure 14:**
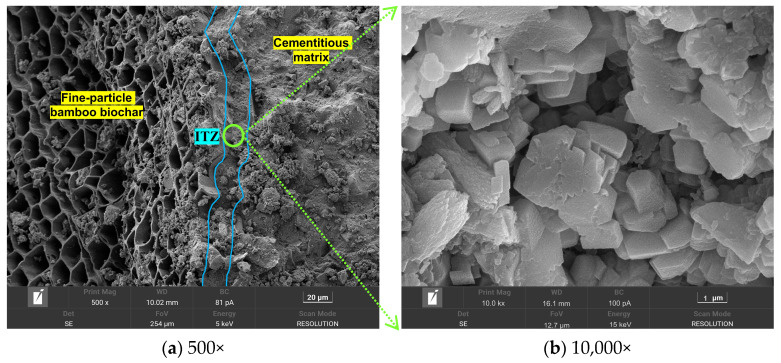
Microstructure morphology of the ITZ between the BB and the shell matrix under CC system.

This phenomenon was effectively improved under the CC system, as shown in Figure 14. The ITZ matrix exhibited a compact texture with few large pores, owing to the reaction of carbon dioxide with the oriented calcium hydroxide crystals accumulating inside the ITZ. The calcium carbonate clusters generated by this reaction effectively filled the ITZ matrix. It is worth noting that incompletely hydrated cement particles were observed in Figure 13a, which may be attributed to the incomplete hydration of the shell material caused by the low water-to-cement ratio strategy employed in the granulation process.

In addition, to further investigate pore distribution and other defects within the artificial aggregates, the aggregate particles were examined using the X-CT technique.

**Figure 15:**
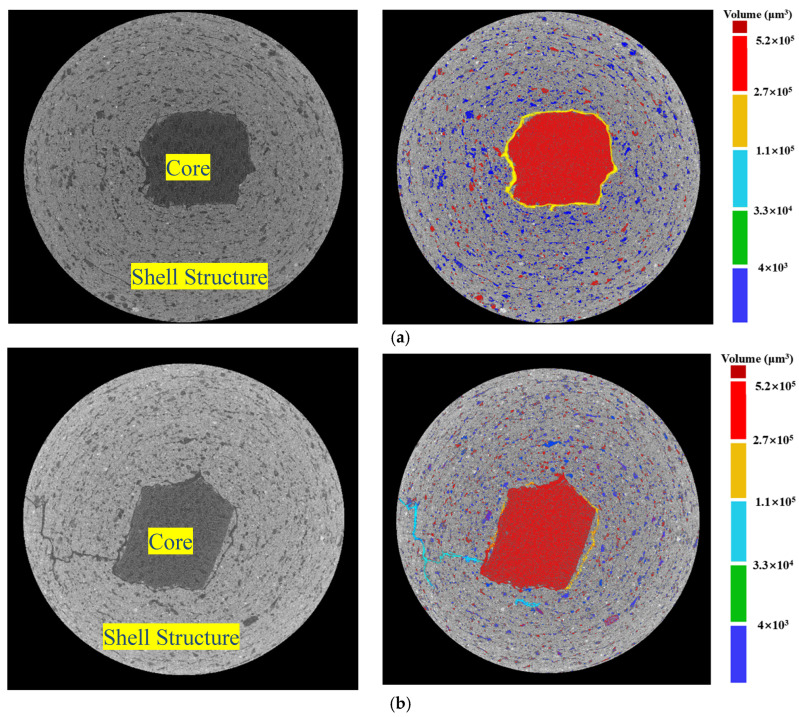
Pore properties of BB-4 group under (**a**) AC system and (**b**) CC system based on X-CT technology.

Figure 15 shows that the shell material matrix contained a large number of small pores with diameters less than 10 μm, in addition to a notable number of large pores with diameters greater than 60 μm. This was due to the incorporation of a certain percentage of F-BB in the shell matrix material. In addition, the ITZ between the core and the shell matrix contained numerous pores with sizes between 20 and 60 μm (yellow area), which was one of the main reasons for the deterioration of bonding ability and mechanical properties of the aggregates.

Compared with the AC system, the pore content in the ITZ between the core and shell matrix in the CC system was lower, as indicated by the smaller yellow region. As shown in Figure 8, carbon dioxide reacted with calcium hydroxide crystals within the shell matrix through the transport channels formed by the internal pores of F-BB particles. The resulting calcium carbonate clusters effectively filled the pores of the shell matrix, as evidenced by the reduction in large pores in the ITZ and the overall decrease in porosity. This conclusion was confirmed in Figure 15.

**Figure 16:**
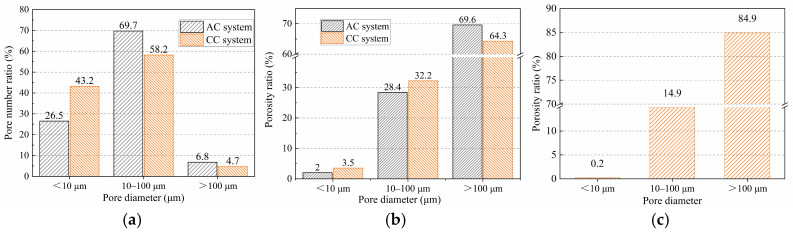
(**a**) pore number ratio, (**b**) porosity ratio, and (**c**) core structure (C-BB) porosity ratio of BB-4 group.

Figure 16 shows the statistical information on aggregate porosity for the BB-4 group. The results show that the proportion of pores smaller than 10 μm in the aggregate shell was 43.2% in the CC system, which was higher than 26.5% in the AC system. The proportions of pores between 10 and 100 μm and greater than 100 μm were 58.2% and 4.7%, respectively, both lower than 69.7% and 6.8% in the AC system. These results indicate that under the CC system, the calcium carbonate clusters generated by the carbonation reaction effectively filled the shell structure, gradually transforming macropores into micropores. In addition, with the gradual consumption of calcium hydroxide, the cement hydration reaction proceeded more completely, further reducing the large pores between the unhydrated cement particles.

### 3.5. CO_2_ Emission

#### 3.5.1. Calculation Method of CO_2_ Emission

The carbon footprint assessment in this study included the carbon emissions associated with raw materials (including production and transportation) and the granulation process (including granulation, transportation, and carbonation) to ensure the accuracy of the calculated emissions from the preparation of artificial aggregates.

In the experiment, 1 ton of artificial aggregates was used as the calculation unit, and all energy consumption involved in the production process was taken into account. The carbon dioxide emission standards for road transportation and electricity consumption referred to the Chinese national standard GB/T 51336-2019 [36], and the carbon emissions of raw materials and various processing procedures were collected from the actual processing operations.

The carbon emissions generated from the production of 1 ton of artificial aggregates were calculated as follows:

(3) SUM=∑i=1nMi∗mci+∑i=1nTi∗di∗mci+γpfe

*M_i_* is the carbon emission factor of materials, including CSW, CCS, biochar, and mixing water. The carbon emission factors of different materials are shown in Table 3. *m_ci_* is the material consumption required to produce 1 ton of artificial aggregates (Table 1). *T_i_* is the carbon emission factor of road transportation, and *d_i_* is the distance that various raw materials were transported from the factory to the aggregate processing site.

In this study, the power of the granulator used for artificial aggregate pelletizing was 5 kW. Based on the statistics of China’s national grid, the carbon emission factor of the electric power carbon footprint was 0.62 kg/kW·h, and the carbon emission factor of pelletizing (γp) was determined to be 3.1 kg CO_2_ eq/h. Considering that the pelletizing process produced 50 kg of artificial aggregates in 29 min, the efficiency of pelletizing per unit of time was determined to be 0.1 t/h.

**Figure 17:**
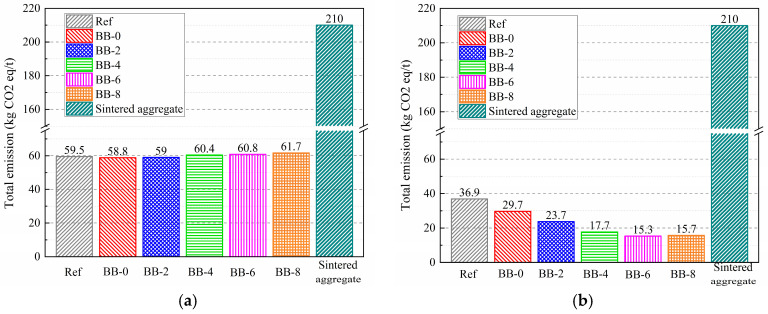
Total carbon emissions per ton of artificial aggregate under (**a**) AC system and (**b**) CC system.

Based on the above accounting rules, the carbon emissions per ton of artificial aggregates were calculated and are presented in Table 4 and Figure 17.

#### 3.5.2. Carbon Emission Calculation and Advantage Assessment

The carbon emissions of artificial aggregates are shown in Figure 17. Under the AC system, the carbon emissions of artificial aggregates were mainly generated from the pelletization process. Taking the Ref group as an example, the production of 1 ton of artificial aggregates produced a total of 59.5 kg of CO_2_ equivalent, of which raw materials, road transportation, and pelletization accounted for 14.7 kg, 14.6 kg, and 30.3 kg, corresponding to proportions of 24.6%, 24.5%, and 50.9%, respectively. These results indicate that the carbon emissions of core–shell aggregates prepared from construction solid waste, industrial solid waste, and biochar were generally reduced by more than 80% compared with cement-based cold-bonded aggregates [37,38], which demonstrates a significant environmental advantage.

Under the CC system, the carbon dioxide absorbed by the carbonation reaction effectively compensated for the carbon emissions generated during the artificial aggregate preparation process, and this advantage became increasingly evident with higher F-BB content. The carbon emissions from the production of 1 ton of artificial aggregates were 29.7 kg, 23.7 kg, 17.7 kg, 15.3 kg, and 15.7 kg at 0%, 2%, 4%, 6%, and 8% F-BB content, respectively. This was attributed to the more complete carbonation of the shell matrix due to the gas-transportation pathways formed by the higher F-BB content.

**Figure 18:**
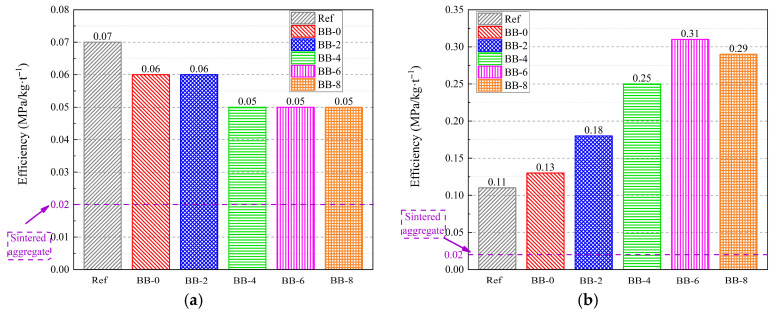
CS/CE ratio of artificial aggregates under (**a**) AC system and (**b**) CC system.

To comprehensively assess the performance advantages of artificial aggregates, the crushing strength–carbon emission ratio (CS/CE) was used to evaluate their engineering properties and environmental benefits. As shown in Figure 18a, the CS/CE values for different groups of artificial aggregates under the AC system were in the range of 0.05–0.07, which was slightly higher than the 0.02 of sintered ceramic aggregates. This indicates that the comprehensive property advantages of core–shell artificial aggregates under the AC system were not significant compared with sintered ceramic aggregates.

As shown in Figure 18b, the CS/CE of artificial aggregates under the CC system increased gradually with higher F-BB content, reaching the highest value of 0.31 when the F-BB content was 6%. Therefore, the BB-6-CC group of artificial aggregates developed in this study is expected to replace sintered ceramic aggregates and conventional cement-based cold-bonded aggregates as building thermal insulation materials due to their excellent engineering properties and reduced carbon emissions.

## 4. Conclusions

Facing the problems of high carbon emissions and shortages of concrete raw materials in the construction industry, this study developed a novel core–shell aggregate preparation method based on the porous characteristics of biochar. It investigated the effects of the curing system and F-BB content on the engineering properties and carbon sequestration potential of artificial aggregates. The following conclusions were drawn:

(1) The loose bulk density of the core–shell lightweight artificial aggregates ranged from 750 to 840 kg/m^3^, which met the engineering requirements for lightweight aggregates. This indicates that the artificial aggregates prepared with biochar possessed the performance advantage of low density.
(2) The incorporation of F-BB not only further reduced the density of artificial aggregates but also formed nearly connected transmission channels of F-BB particles in the shell material matrix, which accelerated the carbonation of calcium hydroxide crystals in the aggregate shell matrix. The carbonation reaction increased the CO_2_ uptake capacity of the core–shell artificial aggregates, which endowed them with both engineering and environmental advantages.
(3) The core–shell artificial aggregates showed promising potential for CO_2_ uptake under the CC system, and when the F-BB content reached 8%, the carbon dioxide uptake per unit mass of the artificial aggregates achieved a 5.78% mass fraction of CO_2_ uptake.
(4) The crushing strength–carbon emission ratio of artificial aggregates in the CC system increased gradually with the increase in F-BB content and reached the highest value of 0.31 when the F-BB content was 6%, which implies that the comprehensive properties of the BB-6-CC group were superior to those of commonly used commercial lightweight aggregates in terms of both engineering performance and carbon emission reduction.

In summary, this paper highlights the property advantages and carbon reduction benefits of core–shell artificial aggregates prepared based on the porous characteristics of bamboo biochar, which provide scientific insights for the efficient utilization of construction and agricultural solid wastes. However, scientific issues such as long-term stability of bamboo biochar under different preparation methods in alkaline environment and volume stability of core–shell artificial aggregates in concrete need to be further explored.

## Figures and Tables

**Table 1:** Chemical composition of CSW and CCS.

Types	CaO	SiO_2_	Al_2_O_3_	Fe_2_O_3_	MgO	SO_3_	TiO_2_	Na_2_O	Others
CSW	35.1	29	9.3	4.5	1.3	2.3	-	0.2	17.2
CCS	68.1	2.2	1.4	0.1	0.3	0.6	-	0.1	27.2

**Table 2:** Mix proportions per cubic meter of fresh artificial aggregate.

Sample	Shell Materials			Core Material (C-BB)	Mixing Water	Curing Methods
	CSW	CCS	F-BB			
Ref-AC	1476	0	0	164	361	AC
BB-0-AC	1328	148	0	164	361	AC
BB-2-AC	1298	148	30	164	386	AC
BB-4-AC	1269	148	59	164	411	AC
BB-6-AC	1239	148	89	164	436	AC
BB-8-AC	1210	148	118	164	460	AC
Ref-CC	1476	0	0	164	361	CC
BB-0-CC	1328	148	0	164	361	CC
BB-2-CC	1298	148	30	164	386	CC
BB-4-CC	1269	148	59	164	411	CC
BB-6-CC	1239	148	89	164	436	CC
BB-8-CC	1210	148	118	164	460	CC

Note: 1. AC and CC represent air curing system and CO_2_ curing system, respectively; 2. Taking BB-4-CC as an example, BB-4 denotes F-BB admixture of 4% (by mass), and CC denotes artificial aggregate cured under CO_2_ curing system; 3. The mass of F-BB added was increased by 2%, and the mixing water was increased by 25 kg.

**Table 3:** Carbon emission factors of raw materials and preparation processes of artificial aggregates.

Raw Materials and Preparation Procedures	Carbon Emission Factor	Source of Data
CSW (Includes cleaning, sedimentation, and filter-pressing processes)	0.008 kg CO_2_ e/kg	A ready-mixed concrete plant in Yantai
CCS	0.006 kg CO_2_ e/kg	An acetylene gas factory in Yantai
BB	0.08 kg CO_2_ e/kg	A biochar plant in Yantai
Mixing water	1.68 × 10^−5^ kg CO_2_ e/kg	GB/T 51336–2019 [36]
Sintered ceramic aggregate	0.21 kg CO_2_ e/kg	A building materials factory in Yantai
Road transportation (2 t class petrol transporter)	0.344 kg CO_2_ e/(t·km)	GB/T 51336–2019 [36]
Pelletization and curing (based on electricity consumption)	3.1 kg CO_2_ e/h	Statistically obtained from the preparation process

Note: ① The transportation distances of CSW, CCS and BB from the plant to the aggregate processing site are 40 km, 35 km and 80 km, respectively. ② The apparent density and crushing strength of Sintered ceramic aggregate are 1680 kg/m^3^ and 4.5 MPa, respectively.

**Table 4:** Carbon emissions from different groups of artificial aggregates (kg/m^3^).

Sample	Raw Materials	Road Transportation	Pelletization	Carbonation	Total
Ref-AC	14.7	14.6	30.3	−0.1	59.5
BB-0-AC	14.5	14.6	29.9	−0.2	58.8
BB-2-AC	15.8	14.2	29.2	−0.2	59
BB-4-AC	17	15	28.6	−0.2	60.4
BB-6-AC	18.2	15.1	27.7	−0.2	60.8
BB-8-AC	19.5	15.4	27	−0.2	61.7
Ref-CC	14.7	14.6	30.3	−22.7	36.9
BB-0-CC	14.5	14.6	29.9	−29.3	29.7
BB-2-CC	15.8	14.2	29.2	−35.5	23.7
BB-4-CC	17	15	28.6	−42.9	17.7
BB-6-CC	18.2	15.1	27.7	−45.7	15.3
BB-8-CC	19.5	15.4	27	−46.2	15.7
Sintered aggregate	-	-	-	-	210

Note: Sintered aggregate is prepared from fly ash as raw material by rotary kiln sintering method (sintering temperature is 900 °C–1100 °C), and the loose bulk density, crushing strength and water absorption of the aggregate are 810 kg/m^3^, 4 MPa and 13.5%, respectively.

## Data Availability

The original contributions presented in this study are included in the article. Further inquiries can be directed to the corresponding author.

## Abbreviations

The following abbreviations are used in this manuscript:

BB	Bamboo biochar
C-BB	Coarse-particle bamboo biochar
CSW	Concrete slurry waste
CCS	Calcium carbide slag
F-BB	Fine-particle bamboo biochar
XRD	X-ray diffraction
SEM	Scanning electron microscopy
X-CT	X-ray computed tomography
AC system	Air curing system
CC system	CO_2_ curing system
C_3_S	Tricalcium silicate
C_2_S	Dicalcium silicate
ITZ	Interfacial transition zone
